# Triglycerides to High-Density Lipoprotein Cholesterol Ratio Is the Best Surrogate Marker for Insulin Resistance in Nonobese Middle-Aged and Elderly Population: A Cross-Sectional Study

**DOI:** 10.1155/2021/6676569

**Published:** 2021-04-30

**Authors:** Yumei Yang, Baomin Wang, Haoyue Yuan, Xiaomu Li

**Affiliations:** Department of Endocrinology and Metabolism, Zhongshan Hospital, Fudan University, No. 180 Fenglin Road, Shanghai 200032, China

## Abstract

**Objective:**

Insulin resistance (IR) is closely associated with metabolic profiles, including obesity and dyslipidemia. The aim of the present study was to examine how lipid profiles were associated with IR in nonobese middle-aged and elderly Chinese population.

**Methods:**

This cross-sectional study included 1608 subjects. IR was defined by homeostasis model assessment of insulin resistance (HOMA-IR) of at least 2.5.

**Results:**

In nonobese subjects (body mass index (BMI) < 25 kg/m^2^, *n* = 996), triglyceride (TG) to high-density lipoprotein cholesterol (HDL-C) ratio (odds ratio (OR) = 1.43, 95% confidence interval (CI) 1.13–1.81, *P*=0.003) was an independent risk factor for IR. The best marker for predicting IR in nonobese subjects was TG/HDL-C ratio with the areas under the receiver operating characteristic curves (AUC) of 0.73 (*P* < 0.001). The optimal cut-off point to identifying IR for TG/HDL-C ratio was ≥1.50 in the nonobese population. Other markers like BMI, TG, and total cholesterol (TC)/HDL-C also had acceptable discriminatory power for predicting IR in nonobese population (AUC ≥ 0.7 and *P* < 0.001). BMI had the highest AUC of 0.647 (*P* < 0.001) after being adjusted, but it was not effective enough to predict IR in obese subjects (BMI ≥ 25.0, *n* = 612).

**Conclusions:**

The TG/HDL-C ratio may be the best reliable marker for predicting IR in the nonobese middle-aged and elderly Chinese population.

## 1. Introduction

Type 2 diabetes mellitus (T2DM) is a worldwide growing health problem. IR and impaired *β*-cell function are considered as the primary defects in T2DM [1]. Currently, the standard methods of measuring IR include the glucose clamp, the modified insulin suppression test, and the HOMA-IR [2–4], but these tests are not routinely measured in most clinical practices owing to the time and cost involved. Thus, early identification of IR by using simple and inexpensive method is essential for preventing T2DM.

IR is characterized by a decrease in the ability of insulin to stimulate the use of glucose by muscles and adipose tissues and to suppress hepatic glucose production and output [5]. It is worth noting that IR is often accompanied by dyslipidemia. Low-density lipoprotein (LDL), HDL, and cholesterol can regulate the function and survival of *β*-cells. HDL even improves insulin sensitivity of muscle and liver. Evidence suggested that dyslipidemias may not be only consequences but also contributors to the pathogenesis of T2DM [6]. McLaughlin et al. first proposed that TG/HDL-C ratio could be used to identify overweight individuals who are insulin-resistant in 2003 [7]. Thereafter, several studies have reported that TG/HDL-C ratio could be a simple marker of IR [3, 4, 8, 9]. However, the relationship between TG/HDL-C ratio and IR differs by ethnicity [3, 10]. The TG/HDL-C ratio may be a good marker to identify insulin-resistant individuals of Aboriginal, Chinese, and European, but not South Asian population [3].

Previous studies in Chinese population have suggested that TG/HDL-C ratio could be a good predictor of IR [4, 8]. But the relationship between TG/HDL-C ratio and IR in nonobese middle-aged and elderly people remains unknown. This study aims to develop a simple predictive marker as a clinical tool for the evaluation of IR in nonobese middle-aged and elderly Chinese population and to further explore the optimal cut-offs.

## 2. Materials and Methods

### 2.1. Ethics, Consent, and Permission

All procedures were carried out in compliance with the Helsinki Declaration. The present study was approved by Zhongshan Hospital ethics committee, Fudan University, China. All the participants signed the informed consent.

### 2.2. Study Population

Participants were continuously enrolled from the Medical Examination Center, Zhongshan Hospital, Fudan University, Shanghai, China, from 2014 to 2016. This cross-sectional study included 1608 (596 men and 1012 women) subjects aged 63 ± 10 years. Healthy adults without prior known diabetes mellitus were included in this study. Exclusion criteria included (1) the presence of diabetes or other severe chronic diseases such as heart and renal diseases, (2) the use of medications that may influence IR or lipid metabolism (such as corticosteroids and lipid-lowering drugs), (3) serious infections and trauma, (4) stress, and (5) the informed consent form not being signed. We used the 1999 World Health Organization criteria to diagnose diabetes patients [11]. Either a fasting plasma glucose ≥7.0 mmol/L or a 2 h postload glucose ≥11.1 mmol/L was defined as diabetes.

### 2.3. General Clinical Data Collection

The patients' demographic data, including age and sex, were obtained from the clinical documents. Anthropometric measures were collected by trained nurses. Body weight (kg) and height (m) were measured while the participants were wearing light clothing and no shoes. The BMI was calculated by weight in kilograms divided by height in meters squared. According to the BMI levels, the subjects were divided into two groups: nonobese (BMI < 25 kg/m^2^) and obese (BMI ≥ 25 kg/m^2^) [12]. Waist circumference (WC) was measured to the nearest 0.1 cm with a tape at the high point of the iliac crest at minimal respiration. Systolic blood pressure (SBP) and diastolic blood pressure (DBP) were measured by a nurse with a mercury sphygmomanometer adapted for arm size after 5 min of rest with the participants in the sitting position. Two blood pressure measurements were recorded at 5 min intervals, and the means were used for the data analysis.

After overnight fasting for 10 h, a 75 g glucose tolerance test was carried out, and blood samples were collected both during fasting and 120 min after administration of the glucose load. Plasma glucose concentration was measured using an enzymatic reaction; a radioimmunoassay method was used to measure serum insulin levels. Standard enzymatic tests were used for fasting lipid profiles (TC, TG, and HDL-C). All the parameters were detected by Hitachi 7180 automatic biochemical analyzer (Tokyo, Japan). LDL-C concentration was calculated as TC minus the cholesterol in the supernatant by the precipitation method using the Friedewald equation [13], non-HDL-C was calculated by subtracting HDL-C from TC, and the TC/HDL-C ratio, TG/HDL-C ratio, and LDL-C/HDL-C ratio were separately calculated. HOMA-IR was calculated as fasting plasma glucose (mmol/L) × fasting insulin (mU/L)/22.5 [14]. IR was defined by HOMA-IR of at least 2.5 [15].

### 2.4. Statistical Analysis

Data are presented as means ± standard deviation (SD) for normal variables or median + interquartile range for skewed variables. Nonnormal values were log-transformed before analysis. Comparisons between groups were performed by Student's *t*-test for normal variables and by the *χ*^2^-test for categorical variables. We analyzed the association of the lipid profiles with IR by logistic regression in different models. The associations of the lipid profiles with IR were analyzed by using multivariate stepwise logistic regression. AUC was used to examine the discriminatory power: AUC of 0.5 = no discrimination, 0.7 ≤ AUC < 0.8 = acceptable discriminative ability, 0.8 ≤ AUC < 0.9 = excellent discriminative ability, AUC ≥ 0.9 = outstanding discriminative ability [8]. The analyses were carried out by SPSS software (version 17.0; SPSS, Chicago, IL, USA).

## 3. Results

The characteristics of the participants are presented in Table 1. Obese people were slightly older than nonobese subjects. Obese individuals have larger WC and higher SBP and DBP than nonobese individuals. Obese individuals have significantly higher TG, TC/HDL-C, and TG/HDL-C than nonobese individuals, while TC and LDL-C levels have no significant difference. In obese subjects, fasting blood glucose, 2 h postprandial blood glucose, INS, and HOMA-IR were significantly higher. TC and LDL-C concentrations did not differ among the groups.

ORs for IR of all subjects are shown in Table 2. Whether in obese or nonobese individuals, dyslipidemia is positively correlated with IR. After adjustments for sex and age, TC/HDL-C (OR = 1.976, 95% CI 1.678–2.326, *P* < 0.001), LDL-C/HDL-C (OR = 1.940, 95% CI 1.524–2.471, *P* < 0.001), and TG/HDL-C (OR = 1.894, 95% CI 1.588–2.259, *P* < 0.001) had significant associations with IR in nonobese subjects. After multivariate adjustments for sex, age, BMI, SBP, and DBP, TC/HDL-C (OR = 1.741, 95% CI 1.434–2.114, *P* < 0.001), LDL-C/HDL-C (OR = 1.618, 95% CI 1.258–2.081, *P* < 0.001), and TG/HDL-C (OR = 1.704, 95% CI 1.420–2.047, *P* < 0.001) had significant associations with IR in nonobese subjects. After multivariate adjustments, the association between the TG/HDL-C ratio and IR was attenuated but remained significant. Among the single lipid markers, the TG levels also showed strong associations with IR in nonobese subjects (OR = 1.917 in model 1 or OR = 1.754 in model 2).

To further investigate the potential independent risk factors for IR among the lipid profiles in nonobese subjects, a multivariate stepwise logistic regression was carried out (Table 3). SBP (OR = 1.012, 95% CI 1.001–1.023, *P*=0.027), TC/HDL-C (OR = 1.482, 95% CI 1.150–1.909, *P*=0.002), and TG/HDL-C (OR = 1.434, 95% CI 1.132–1.816, *P*=0.003) were independently associated with IR in nonobese subjects. Namely, for a 1-unit increase in TG/HDL-C, the odds of being insulin-resistant increased 1.434 times in nonobese individuals.

**Figure 1:**
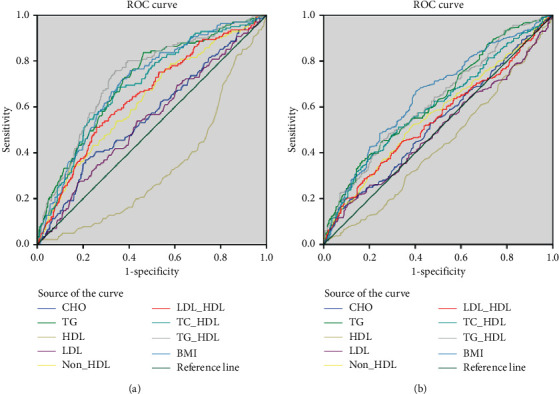
ROC curves of TG/HDL-C and other lipid components or markers of insulin resistance. ROC curves of TC, TG, HDL-C, LDL-C, non-HDL-C, LDL-C/HDL-C, TC/HDL-C, TG/HDL-C, and BMI were presented in nonobese and obese subjects. (a) ROC curves in nonobese subjects. (b) ROC curves in obese subjects.

The AUC for potential markers of IR are presented in Table 4, Figure 1, and Supplementary Figures. After being adjusted for sex and age, the AUC for BMI, TG, HDL-C, non-HDL-C, LDL-C/HDL-C, TC/HDL-C, TG/HDL-C, and TG minus HDL-C to predict IR were 0.712, 0.711, 0.689, 0.630, 0.652, 0.700, 0.732, and 0.729 (all *P* values are less than 0.001) in nonobese subjects, respectively. Before being adjusted, the TG/HDL-C ratio had an AUC of 0.728 (*P* < 0.001) for predicting IR. The AUC value improved to 0.732 (*P* < 0.001) after being adjusted for sex and age, which indicated that the TG/HDL-C ratio can predict IR independent of sex and age. Other markers like BMI, TG, and TC/HDL-C also had acceptable discriminatory power for predicting IR in nonobese population (AUC ≥ 0.7 and *P* < 0.001). These markers had slightly elevated AUC values after being adjusted for sex and age, which suggested that BMI, TG, TC/HDL-C, TG/HDL-C, and TG minus HDL-C can predict IR independent of sex and age in nonobese subjects. After being adjusted for sex and age, TG minus HDL-C had an AUC of 0.729 (*P* < 0.001) for predicting IR in nonobese subjects, which was weaker than the TG/HDL-C ratio (AUC 0.732, *P* < 0.001). However, none of these markers were acceptable to predict IR in obese subjects before and after being adjusted for covariates. BMI had the highest AUC of 0.647 (*P* < 0.001) after being adjusted, but it was not effective enough to predict IR in obese subjects.

The best marker for prediction of IR in nonobese subjects was TG/HDL-C ratio with the AUC of 0.728, and BMI had an AUC of 0.705 for predicting IR. The difference between these two markers was not significant (*P*=0.448, DeLong's test). However, with the addition of BMI, the AUC of the TG/HDL-C ratio improved to 0.760 (*P* < 0.001, DeLong's test), which is statistically significant. The AUC of BMI and the TG/HDL-C ratio in obese subjects are 0.647 and 0.633, respectively. The difference of their discriminatory power is not statistically significant (*P*=0.464, DeLong's test). The AUC of the TG/HDL-C ratio improved to 0.691 after adding BMI (*P* < 0.001, DeLong's test). These results suggested that TG/HDL-C combined with BMI could predict IR better.

## 4. Discussion

The findings of our study indicate that the TG/HDL-C ratio is strongly associated with IR in nonobese middle-aged and elderly Chinese people. Our study has not found a good marker for predicting IR in obese subjects. The TG/HDL-C ratio may be a better marker to predict IR than BMI, but the difference was not statistically significant due to our limited sample size. TG/HDL-C combined with BMI could predict IR better.

There are conflicting data on the association of TG/HDL ratio with IR among different populations. Previous studies have reported different cut-off values of TG/HDL-C ratio to detect the presence of IR. McLaughlin et al. first proposed TG/HDL-C to identify overweight individuals who are insulin-resistant in 2003 [7].This cross-sectional study included 258 nondiabetic overweight or obese individuals, of whom 87% were non-Hispanic whites. They reported that a cut-off value of TG/HDL-C ratio of 3.0 could reliably predict IR in overweight people. Furthermore, only 129 of the overweight or obese persons were identified as insulin-resistant (positive predictive value, 50%), which means not all overweight or obese people are insulin-resistant. In fact, resistance to insulin-mediated glucose disposal is distributed continuously throughout the general population [16]. This conclusion emphasized the importance of early identification of high-risk individuals in nonobese population. One year later, a study demonstrated that the TG/HDL-C ratio only positively correlates with IR in severely obese nondiabetic individuals but not in patients with overt diabetes [17]. Racial/ethnic differences in triglyceride concentrations and HDL-C values have been widely reported. A study found that non-Hispanic blacks had lower TG concentrations than non-Hispanic whites or Mexican Americans [18]. The authors proposed race/ethnicity-specific TG/HDL-C cut-off points to predict IR: cut-off points of 3.0 for non-Hispanic whites and Mexican Americans and 2.0 for non-Hispanic blacks. Moreover, the findings of their study suggested that the association of the TG/HDL-C ratio with hyperinsulinemia was stronger among people with a BMI <25 kg/m^2^ than those with a BMI ≥ 30 kg/m^2^. As reported in a previous study, about 16% of people with normal weight (BMI < 25 kg/m^2^) were identified to be insulin-resistant [19]. This result encourages us to establish a simple and useful method to detect IR individuals among nonobese people. However, a cross-sectional study in overweight African Americans has reported that the triglyceride or TG/HDL-C ratio was not significantly associated with IR. The AUC value of the TG/HDL-C ratio to predict IR was 0.56, which is not significant [20].

Currently, there is no consensus on the best marker for prediction of IR in nonobese Asian population. He et al. reported that TG/HDL-C could discriminate IR in the nonobese and normoglycaemic Chinese women, with the AUC of 0.718. Their findings showed that the discriminatory power of TG/HDL-C for IR differs by genders and BMI in Chinese population. The discriminatory power of TG/HDL-C for IR was only acceptable in the nonobese women, but not acceptable in the obese women and men [8]. Liver markers could also predict IR. A study demonstrated that ALT/AST ratio may be the best reliable marker of IR in nonobese Japanese adults. ALT/AST ratio of ≥0.82 in nonobese subjects and ≥1.02 in overweight subjects could predict IR effectively in Japanese population [21]. However, Sun et al. investigated the association of liver enzymes and lipid profiles with IR in middle-aged and older nonobese Chinese without diabetes and found that the TG/HDL-C ratio is better than liver enzymes to identify IR in middle-aged and older nonobese Chinese adults [22]. Although there is no agreement on the best marker for predicting IR, a majority of studies based on Chinese population demonstrated that TG/HDL-C has great discriminatory power to predict IR [4, 8, 23].

It is worth noting that TG/HDL-C has also been shown to predict cardiovascular events and nonalcoholic fatty liver disease (NAFLD) independently [24, 25]. In addition to being associated with IR, the lipoprotein phenotype is also associated with increased cardiovascular disease. A study of McLaughlin's group showed that TG/HDL ratio ≥3.5 predicts the presence of the small dense LDL phenotype (LDL phenotype B) with high sensitivity and specificity [24]. Those insulin-resistant, dyslipidemic patients are at increased risk of cardiovascular disease. Cystatin C has been found to be closely related to T2DM [26] and coronary heart disease [27]. Recently, Klisic et al. reported that serum cystatin C levels were associated with TG/HDL-C ratio in adolescent girls [28], suggesting TG/HDL-C could be a surrogate marker of cardiometabolic disease. A cross-sectional study reported that a cut-off value of TG/HDL-C ratio of 0.9 in women (sensitivity = 78.8%, specificity = 77.3%) and 1.4 in men (sensitivity = 70.7%, specificity = 73.5%) could predict NAFLD.

The mechanism of TG/HDL-C predicting IR is still unclear. When circulating TG persists at high levels, heparin activates lipoprotein lipase to increase intravascular lipolysis of TG, thus increasing the risk of tissue exposure to free fatty acids (FFAs). High FFAs may deteriorate insulin sensitivity via oxidative stress pathway [29, 30]. Previous studies have indicated that the TG/HDL-C ratio as a marker of lipotoxicity in *β*-cells results in impaired insulin secretion [31] and increased *β*-cell apoptosis from high circulating TG levels [32]. Shimabukuro et al. demonstrated that *β*-cell apoptosis is induced by increased FFA via de novo ceramide formation and increased nitric oxide (NO) production. Elevated levels of circulating FFA and lipoproteins transport to islets far more FFA than can be oxidized, leading to an increase in ceramide, inducible nitric oxide synthase (iNOS) expression, and NO production, which cause apoptosis [32].

After being adjusted for sex and age, TG minus HDL-C had an AUC of 0.729 (*P* < 0.001) for predicting IR in nonobese subjects, which was weaker than the TG/HDL-C ratio (AUC 0.732, *P* < 0.001). Previous study has demonstrated that plasma triglyceride and HDL-C levels are independently associated with IR. McLaughlin et al. first proposed that TG to HDL-C ratio could be used to identify overweight individuals who are insulin-resistant in 2003 [7]. After that, many studies reported that the TG/HDL-C ratio was a surrogate marker of IR [3, 8, 10]. The TG/HDL-C ratio has several advantages. First of all, lipid concentrations are commonly available through standard measurements, so it could be widely used. Besides, as we mentioned before, increased TG/HDL-C ratios also indicate the presence of atherogenic small, dense LDL particles and could serve as a good predictor of myocardial infarction and the presence of coronary atherosclerotic lesions. LDL subclass phenotype B, characterized by a predominance of small dense LDL, is an integral feature of the IR syndrome. The TG/HDL-C ratio ≥3.5 can predict the presence of the small dense LDL phenotype (LDL phenotype B) with high sensitivity and specificity [24]. On the contrary, there are no related studies on TG minus HDL-C so far. This marker with uncertain meaning may deserve further research in the future.

Our study demonstrated that TG/HDL-C could only predict IR in nonobese population. In obese subjects, BMI had the highest AUC (AUC = 0.648, *P* < 0.001) for predicting IR, and TG had the highest AUC (AUC = 0.628, *P* < 0.001) for predicting IR among lipid markers. The discriminatory power of these two markers was not acceptable. As the average levels of blood pressure and blood glucose of obese population were significantly higher than nonobese population, the discriminative ability of lipid profile markers might be underestimated. In addition, the sample size of obese population was limited, which could restrict the discriminative ability of lipid profile markers. Previous researches have reported several predictors for IR in obese population, such as sagittal abdominal diameter [33], Fetuin-A [34], and triglyceride/glucose index [35]. Further research should be done to explore a lipid marker for predicting IR in obese population.

There are several limitations in our study. First, the major limitation of this study was our failure to use a glucose clamp, an insulin suppression test, or the frequently sampled intravenous glucose tolerance test. However, we used fasting insulin concentration and HOMA-IR to demonstrate IR, which is practical in clinical settings. Second, because of the relatively small sample size, the results of our study might have limited statistical power. Third, this study was a cross-sectional design that made it difficult to establish the causal relationship between TG/HDL-C and IR. No comparisons between different races might be another limitation. We would conduct validation or calibration to prove robustness of TG/HDL-C ratio in the future to make the results of our study be more broadly applicable.

## 5. Conclusion

In conclusion, our findings demonstrated that the elevated TG/HDL-C ratio was significantly associated with IR and could be used as an indicator of IR among the nonobese middle-aged and elderly Chinese population. TG/HDL-C ratio could be recommended in clinical work to early identify insulin-resistant patients, and interventions such as lifestyle changes could be taken.

## Figures and Tables

**Table 1:** Clinical and biochemical characteristics of the study subjects according to BMI.

Variables	All (*n* = 1608)	Nonobese (BMI < 25 kg/m^2^)	Obese (BMI ≥ 25 kg/m^2^)	*P* value
Age (years)	63.18 ± 10.24	62.74 ± 10.30	63.90 ± 10.10	0.027
Sex (M/F)	596/1012	349/647	246/365	0.035
BMI	24.18 ± 3.33	22.13 ± 1.92	27.54 ± 2.25	<0.001
WC (cm)	83.07 ± 9.69	78.05 ± 6.87	91.27 ± 7.87	<0.001
SBP (mmHg)	134.88 ± 19.03	131.40 ± 18.66	140.60 ± 18.23	<0.001
DBP (mmHg)	76.37 ± 10.68	74.64 ± 10.42	79.19 ± 10.52	<0.001
TC (mmol/L)	5.13 ± 0.93	5.14 ± 0.93	5.11 ± 0.92	0.475
TG (mmol/L)	1.69 ± 1.11	1.52 ± 1.03	1.97 ± 1.18	<0.001
LDL-C (mmol/L)	2.97 ± 0.79	2.98 ± 0.79	2.94 ± 0.78	0.283
HDL-C (mmol/L)	1.41 ± 0.36	1.48 ± 0.37	1.30 ± 0.31	<0.001
Non-HDL-C (mmol/L)	3.72 ± 0.88	3.66 ± 0.90	3.81 ± 0.85	0.001
TC/HDL-C	3.81 ± 1.01	3.64 ± 1.00	4.09 ± 0.95	<0.001
TG/HDL-C	1.36 ± 1.15	1.16 ± 1.02	1.69 ± 1.26	<0.001
LDL-C/HDL-C	2.21 ± 0.75	2.13 ± 0.77	2.35 ± 0.70	<0.001
FBG (mmol/L)	5.26 ± 1.01	5.10 ± 0.69	5.52 ± 1.33	<0.001
2hPG (mmol/L)	7.59 ± 3.31	6.99 ± 2.79	8.57 ± 3.81	<0.001
INS	7.80 (5.30–11.30)	6.40 (4.50–9.01)	11.10 (7.80–14.79)	<0.001
HOMA-IR	1.78 (1.19–2.66)	1.44 (0.98–2.07)	2.56 (1.78–3.63)	<0.001

Data are presented as means ± SD, median (interquartile range), or percentage.

**Table 2:** The risk of insulin resistance according to lipid profiles.

	All				Nonobese				Obese			
	Model 1		Model 2		Model 1		Model 2		Model 1		Model 2	
	OR (95% CI)	*P*	OR (95% CI)	*P*	OR (95% CI)	*P*	OR (95% CI)	*P*	OR (95% CI)	*P*	OR (95% CI)	*P*
TC (mmol/L)	1.154 (1.021–1.304)	0.021	1.196 (1.042–1.373)	0.011	1.233 (1.006–1.488)	0.044	1.178 (0.964–1.439)	0.110	1.173 (0.976–1.409)	0.089	1.192 (0.984–1.443)	0.073
TG (mmol/L)	2.006 (1.764–2.281)	<0.001	1.715 (1.499–1.961)	<0.001	1.917 (1.590–2.312)	<0.001	1.754 (1.442–2.133)	<0.001	1.626 (1.353–1.953)	<0.001	.631 (1.352–1.969)	<0.001
LDL-C (mmol/L)	1.022 (0.888–1.177)	0.759	1.029 (0.879–1.206)	0.721	1.138 (0.909–1.425)	0.260	1.047 (0.829–1.321)	0.700	0.991 (0.804–1.221)	0.932	1.001 (0.806–1.243)	0.993
HDL-C (mmol/L)	0.133 (0.090–0.197)	<0.001	0.250 (0.161–0.388)	<0.001	0.127 (0.068–0.237)	<0.001	0.197 (0.103–0.379)	<0.001	0.336 (0.187–0.603)	<0.001	0.348 (0.189–0.640)	0.001
Non-HDL-C (mmol/L)	1.497 (1.318–1.700)	<0.001	1.420 (1.231–1.639)	<0.001	1.576 (1.296–1.917)	<0.001	1.426 (1.163–1.749)	0.001	1.350 (1.109–1.643)	0.003	1.371 (1.117–1.684)	0.003
LDL-C/HDL-C	1.762 (1.507–2.059)	<0.001	1.488 (1.252–1.768)	<0.001	1.940 (1.524–2.471)	<0.001	1.618 (1.258–2.081)	<0.001	1.318 (1.041–1.670)	0.022	1.329 (1.039–1.699)	0.023
TC/HDL-C	1.907 (1.706–2.132)	<0.001	1.672 (1.462–1.912)	<0.001	1.976 (1.678–2.326)	<0.001	1.741 (1.434–2.114)	<0.001	1.505 (1.268–1.787)	<0.001	1.564 (1.292–1.893)	<0.001
TG/HDL-C	1.927 (1.711–2.171)	<0.001	1.650 (1.458–1.867)	<0.001	1.894 (1.588–2.259)	<0.001	1.704 (1.420–2.047)	<0.001	1.544 (1.307–1.823)	<0.001	1.560 (1.316–1.848)	<0.001

Data are odds ratios (95% confidence interval); model 1 is adjusted for age and sex; model 2 is further adjusted for BMI, SBP, and DBP.

**Table 3:** Multiple logistic regression analysis: the associations of SBP, lipid ratios, and insulin resistance.

	Nonobese		Obese	
	OR (95% CI)	*P*	OR (95%CI)	*P*
SBP (mmHg)	1.012 (1.001–1.023)	0.027	1.015 (1.005–1.025)	0.003
TC/HDL-C	1.482 (1.150–1.909)	0.002	/^*∗*^	/^*∗*^
TG/HDL-C	1.434 (1.132–1.816)	0.003	1.507 (1.275–1.782)	<0.001

^*∗*^Not statistically significant.

**Table 4:** Area under receiver operating characteristic curves for potential markers of insulin resistance before and after being adjusted for sex and age.

	Nonobese				Obese			
	Crude		Adjusted		Crude		Adjusted	
	AROC	*P*	AROC	*P*	AROC	*P*	AROC	*P*
BMI	0.705	<0.001	0.712	<0.001	0.648	<0.001	0.647	<0.001
TC (mmol/L)	0.563	0.017	0.599	0.028	0.532	0.167	0.530	0.195
TG (mmol/L)	0.712	<0.001	0.711	<0.001	0.628	<0.001	0.631	<0.001
HDL-C (mmol/L)	0.664	<0.001	0.689	<0.001	0.569	0.003	0.588	<0.001
LDL-C (mmol/L)	0.545	0.092	0.539	0.151	0.499	0.978	0.505	0.821
Non-HDL-C (mmol/L)	0.631	<0.001	0.630	<0.001	0.566	0.005	0.565	0.005
LDL-C/HDL-C	0.648	<0.001	0.652	<0.001	0.547	0.043	0.552	0.025
TC/HDL-C	0.696	<0.001	0.700	<0.001	0.604	<0.001	0.612	<0.001
TG/HDL-C	0.728	<0.001	0.732	<0.001	0.624	<0.001	0.633	<0.001
TG minus HDL-C	0.727	<0.001	0.729	<0.001	0.623	<0.001	0.631	<0.001

## Data Availability

The datasets used and/or analyzed during the current study are available from the corresponding author on reasonable request.

## Supplementary Materials

Supplementary Materials Suppl Figure 1: ROC curves of potential markers for predicting insulin resistance. (A–J) ROC curves of BMI, TC, TG, HDL-C, LDL-C, non-HDL-C, LDL-C/HDL-C, TC/HDL-C, TG/HDL-C, and TG minus HDL-C in nonobese subjects. Green line: crude estimation; blue line: adjusted for age and sex. Suppl Figure 2: ROC curves of potential markers for predicting insulin resistance. (A–J) ROC curves of BMI, TC, TG, HDL-C, LDL-C, non-HDL-C, LDL-C/HDL-C, TC/HDL-C, TG/HDL-C, and TG minus HDL-C in obese subjects. Green line: crude estimation; blue line: adjusted for age and sex. Suppl Figure 3: ROC curves of internal validation. (A) 80% nonobese subjects for proposed model. (B) 20% nonobese subjects for internal validation. (C) 80% obese subjects for proposed model. (D) 20% obese subjects for internal validation. Suppl Table 1: clinical and biochemical characteristics of the study subjects according to BMI. Suppl Table 2: the risk of insulin resistance according to lipid profiles. Suppl Table 3: internal validation (80% for proposed model and 20% for internal validation)
