# Supplementary material for: Triglycerides to High-Density Lipoprotein Cholesterol Ratio Is the Best Surrogate Marker for Insulin Resistance in Nonobese Middle-Aged and Elderly Population: A Cross-Sectional Study
Source: Int J Endocrinol. 2021 Apr 30;2021:6676569. doi: 10.1155/2021/6676569 (PMC8110426; doi:10.1155/2021/6676569)
Supplement: Supplementary Materials — Suppl Figure 1: ROC curves of potential markers for predicting insulin resistance. (A–J) ROC curves of BMI, TC, TG, HDL-C, LDL-C, non-HDL-C, LDL-C/HDL-C, TC/HDL-C, TG/HDL-C, and TG minus HDL-C in nonobese subjects. Green line: crude estimation; blue line: adjusted for age and sex. Suppl Figure 2: ROC curves of potential markers for predicting insulin resistance. (A–J) ROC curves of BMI, TC, TG, HDL-C, LDL-C, non-HDL-C, LDL-C/HDL-C, TC/HDL-C, TG/HDL-C, and TG minus HDL-C in obese subjects. Green line: crude estimation; blue line: adjusted for age and sex. Suppl Figure 3: ROC curves of internal validation. (A) 80% nonobese subjects for proposed model. (B) 20% nonobese subjects for internal validation. (C) 80% obese subjects for proposed model. (D) 20% obese subjects for internal validation. Suppl Table 1: clinical and biochemical characteristics of the study subjects according to BMI. Suppl Table 2: the risk of insulin resistance according to lipid profiles. Suppl Table 3: internal validation (80% for proposed model and 20% for internal validation) [file 6676569.f1.zip › 6676569.f1/Supplementary Figures (1).docx]

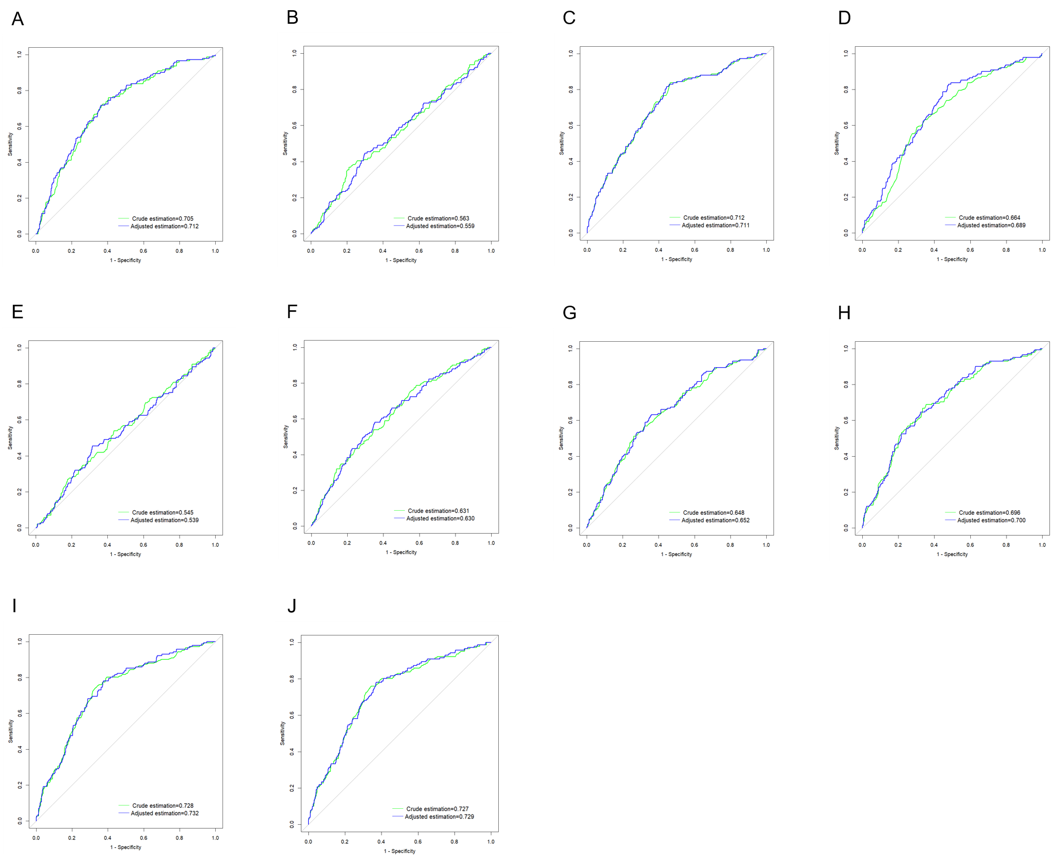


**Suppl Fig 1** **ROC curves of potential markers for predicting insulin resistance.** A-J. ROC curves of BMI, TC, TG, HDL-C, LDL-C, Non-HDL-C, LDL-C/HDL-C, TC/HDL-C, TG/HDL-C and TG minus HDL-C in non-obese subjects. Green line: Crude estimation; Blue line: Adjusted for age and sex.


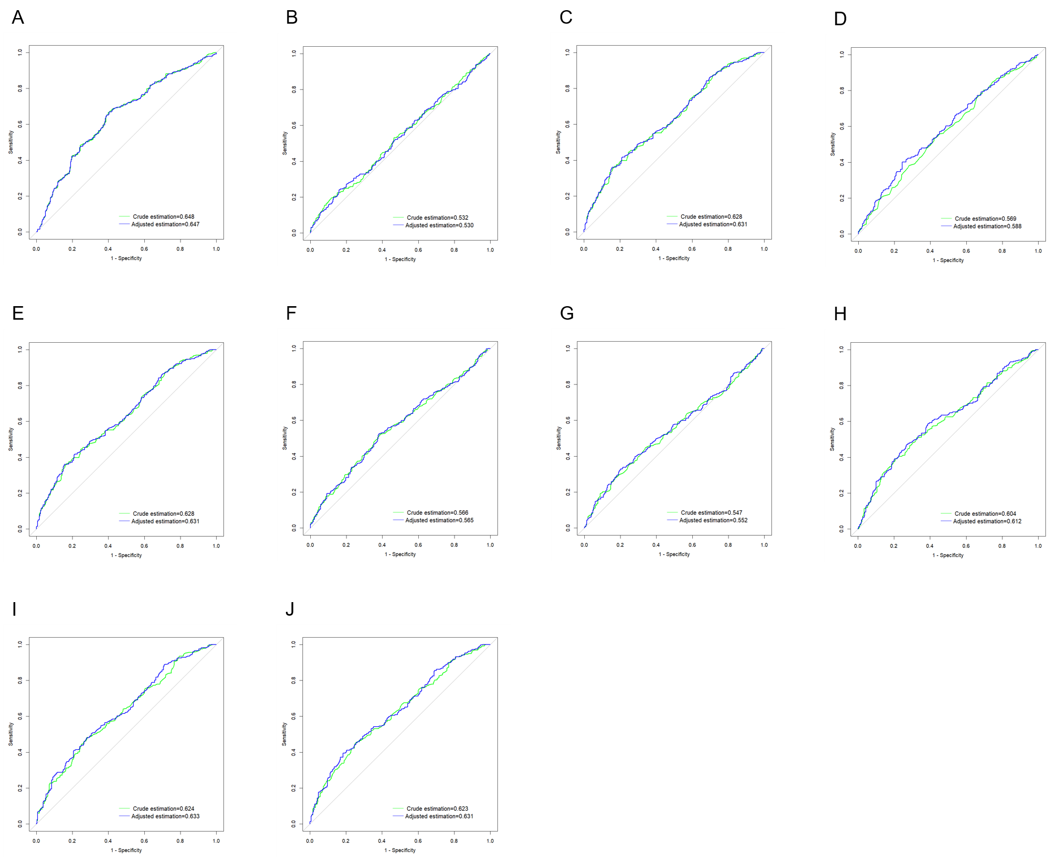


**Suppl Fig 2** **ROC curves of potential markers for predicting insulin resistance.** A-J. ROC curves of BMI, TC, TG, HDL-C, LDL-C, Non-HDL-C, LDL-C/HDL-C, TC/HDL-C, TG/HDL-C and TG minus HDL-C in obese subjects. Green line: Crude estimation; Blue line: Adjusted for age and sex.


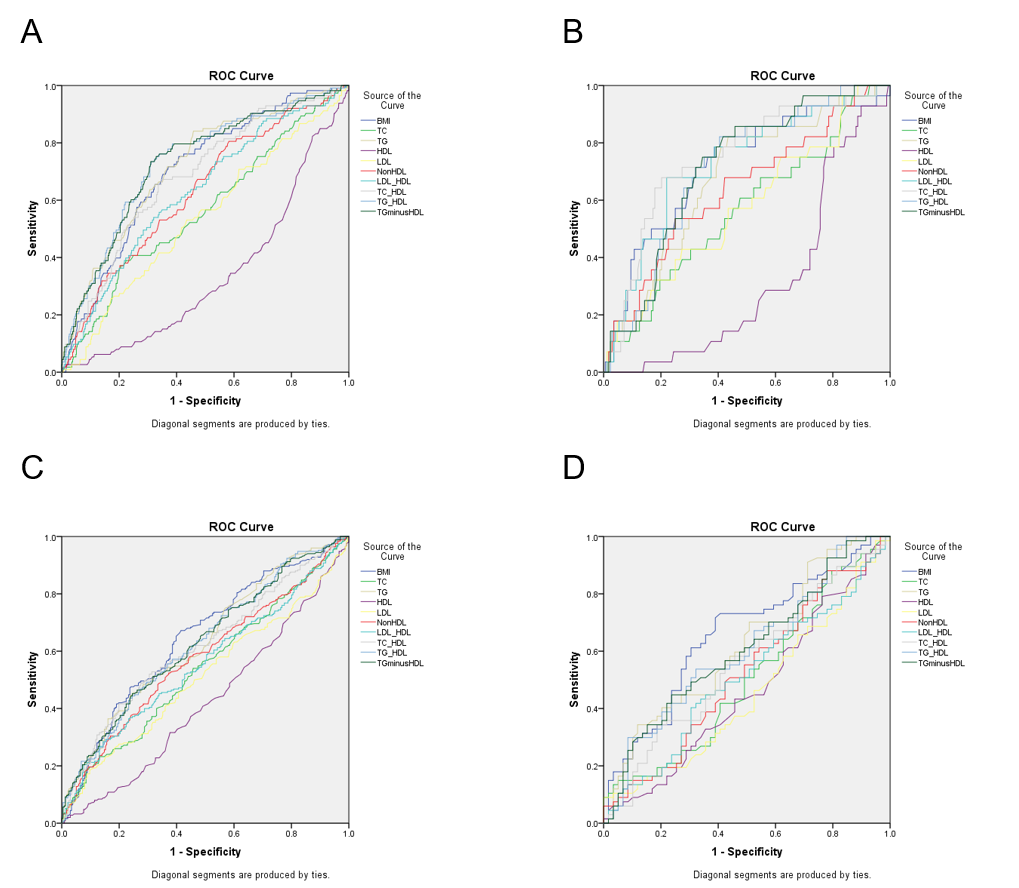


**Suppl Fig 3** **ROC curves of internal validation.** A. 80% non-obese subjects for proposed model. B. 20% non-obese subjects for internal validation. C. 80% obese subjects for proposed model. B. 20% obese subjects for internal validation.
