# Supplementary material for: Triglycerides to High-Density Lipoprotein Cholesterol Ratio Is the Best Surrogate Marker for Insulin Resistance in Nonobese Middle-Aged and Elderly Population: A Cross-Sectional Study
Source: Int J Endocrinol. 2021 Apr 30;2021:6676569. doi: 10.1155/2021/6676569 (PMC8110426; doi:10.1155/2021/6676569)
Supplement: Supplementary Materials — Suppl Figure 1: ROC curves of potential markers for predicting insulin resistance. (A–J) ROC curves of BMI, TC, TG, HDL-C, LDL-C, non-HDL-C, LDL-C/HDL-C, TC/HDL-C, TG/HDL-C, and TG minus HDL-C in nonobese subjects. Green line: crude estimation; blue line: adjusted for age and sex. Suppl Figure 2: ROC curves of potential markers for predicting insulin resistance. (A–J) ROC curves of BMI, TC, TG, HDL-C, LDL-C, non-HDL-C, LDL-C/HDL-C, TC/HDL-C, TG/HDL-C, and TG minus HDL-C in obese subjects. Green line: crude estimation; blue line: adjusted for age and sex. Suppl Figure 3: ROC curves of internal validation. (A) 80% nonobese subjects for proposed model. (B) 20% nonobese subjects for internal validation. (C) 80% obese subjects for proposed model. (D) 20% obese subjects for internal validation. Suppl Table 1: clinical and biochemical characteristics of the study subjects according to BMI. Suppl Table 2: the risk of insulin resistance according to lipid profiles. Suppl Table 3: internal validation (80% for proposed model and 20% for internal validation) [file 6676569.f1.zip › 6676569.f1/Supplementary Tables (2).docx]

**Suppl Table 1**|Clinical and biochemical characteristics of the study subjects according to BMI.

| Variables | All(n=1608) | Non-obese  (BMI<25) | Obese  (BMI≥25) | *P*-value |
| --- | --- | --- | --- | --- |
| TG minus HDL-C | 0.28±1.29 | 0.04±1.21 | 0.67±1.33 | <0.001 |

Data are presented as means ± SD, median (interquartile range) or percentage.

| **Suppl Table 2**\| The risk of insulin resistance according to lipid profiles. | Obese | Model 2 | p | <0.001 | Data are odds ratios (95% confidence interval); Model 1 is adjusted for age, sex; Model 2 is further adjusted for BMI, SBP and DBP. |
| --- | --- | --- | --- | --- | --- |
|  |  |  | OR (95% CI) | 1.545(1.316-1.813) |  |
|  |  | Model 1 | p | <0.001 |  |
|  |  |  | OR (95%CI) | 1.543(1.320-1.804) |  |
|  | Non-obese | Model 2 | p | <0.001 |  |
|  |  |  | OR (95%CI) | 1.686(1.428-1.992) |  |
|  |  | Model 1 | P | <0.001 |  |
|  |  |  | OR (95%CI) | 1.845(1.574-2.163) |  |
|  | All | Model 2 | p | <0.001 |  |
|  |  |  | OR (95%CI) | 1.634(1.457-1.832) |  |
|  |  | Model 1 | P | <0.001 |  |
|  |  |  | OR (95%CI) | 1.902(1.707-2.119) |  |
|  |  |  |  | TG minus HDL-C |  |

**Suppl Table 3**| Internal validation (80% for proposed model and 20% for internal validation).

|  | Non-obese | | | | Obese | | | |
| --- | --- | --- | --- | --- | --- | --- | --- | --- |
|  | Proposed model | | Internal validation | | Proposed model | | Internal validation | |
|  | AROC | p | AROC | p | AROC | p | AROC | p |
| BMI (kg/m^2)^ | 0.701 | 0.025 | 0.718 | 0.054 | 0.645 | 0.025 | 0.658 | 0.049 |
| TC (mmol/L) | 0.563 | 0.030 | 0.566 | 0.059 | 0.540 | 0.026 | 0.505 | 0.052 |
| TG (mmol/L) | 0.720 | 0.025 | 0.677 | 0.052 | 0.631 | 0.025 | 0.622 | 0.050 |
| HDL-C (mmol/L) | 0.341 | 0.027 | 0.315 | 0.046 | 0.424 | 0.026 | 0.462 | 0.052 |
| LDL-C (mmol/L) | 0.537 | 0.030 | 0.576 | 0.059 | 0.511 | 0.026 | 0.455 | 0.052 |
| Non-HDL-C (mmol/L) | 0.630 | 0.028 | 0.639 | 0.058 | 0.576 | 0.026 | 0.523 | 0.052 |
| LDL-C/HDL-C | 0.630 | 0.028 | 0.724 | 0.052 | 0.556 | 0.026 | 0.510 | 0.052 |
| TC/HDL-C | 0.684 | 0.027 | 0.748 | 0.050 | 0.615 | 0.025 | 0.561 | 0.051 |
| TG/HDL-C | 0.733 | 0.026 | 0.710 | 0.047 | 0.628 | 0.025 | 0.608 | 0.050 |
| TG minus HDL-C | 0.730 | 0.026 | 0.714 | 0.047 | 0.627 | 0.025 | 0.609 | 0.050 |
